# Constructing a prognostic model for colorectal cancer with synchronous liver metastases after preoperative chemotherapy: a study based on SEER and an external validation cohort

**DOI:** 10.1007/s12094-024-03513-5

**Published:** 2024-06-04

**Authors:** Yixin Ding, Xiaoxi Han, Shufen Zhao, Shasha Wang, Jing Guo, Chuanyu Leng, Xiangxue Li, Kongjia Wang, Wensheng Qiu, Weiwei Qi

**Affiliations:** 1 Department of Oncology, The Affiliated Hospital of Qingdao University, Qingdao, China; 2 Department of Urology, Qingdao Municipal Hospital, Qingdao University, Qingdao, China; 3 Department of Medical Oncology, Department of Cancer Center, Peking Union Medical College Hospital, Chinese Academy of Medical Sciences, Beijing, China

**Keywords:** Synchronous CRLM, Preoperative chemotherapy, Prognosis, Risk model

## Abstract

**Background:**

The combination of preoperative chemotherapy and surgical treatment has been shown to significantly enhance the prognosis of colorectal cancer with liver metastases (CRLM) patients. Nevertheless, as a result of variations in clinicopathological parameters, the prognosis of this particular group of patients differs considerably. This study aimed to develop and evaluate Cox proportional risk regression model and competing risk regression model using two patient cohorts. The goal was to provide a more precise and personalized prognostic evaluation system.

**Methods:**

We collected information on individuals who had a pathological diagnosis of colorectal cancer between 2000 and 2019 from the Surveillance, Epidemiology, and End Results (SEER) Database. We obtained data from patients who underwent pathological diagnosis of colorectal cancer and got comprehensive therapy at the hospital between January 1, 2010, and June 1, 2022. The SEER data collected after screening according to the inclusion and exclusion criteria were separated into two cohorts: a training cohort (training cohort) and an internal validation cohort (internal validation cohort), using a random 1:1 split. Subgroup Kaplan–Meier (K–M) survival analyses were conducted on each of the three groups. The data that received following screening from the hospital were designated as the external validation cohort. The subsequent variables were chosen for additional examination: age, gender, marital status, race, tumor site, pretreatment carcinoembryonic antigen level, tumor size, T stage, N stage, pathological grade, number of tumor deposits, perineural invasion, number of regional lymph nodes examined, and number of positive regional lymph nodes. The primary endpoint was median overall survival (mOS). In the training cohort, we conducted univariate Cox regression analysis and utilized a stepwise regression approach, employing the Akaike information criterion (AIC) to select variables and create Cox proportional risk regression models. We evaluated the accuracy of the model using calibration curve, receiver operating characteristic curve (ROC), and area under curve (AUC). The effectiveness of the models was assessed using decision curve analysis (DCA). To evaluate the non-cancer-related outcomes, we analyzed variables that had significant impacts using subgroup cumulative incidence function (CIF) and Gray’s test. These analyses were used to create competing risk regression models. Nomograms of the two models were constructed separately and prognostic predictions were made for the same patients in SEER database.

**Results:**

This study comprised a total of 735 individuals. The mOS of the training cohort, internal validation cohort, and QDU cohort was 55.00 months (95%CI 46.97–63.03), 48.00 months (95%CI 40.65–55.35), and 68.00 months (95%CI 54.91–81.08), respectively. The multivariate Cox regression analysis revealed that age, N stage, presence of perineural infiltration, number of tumor deposits and number of positive regional lymph nodes were identified as independent prognostic risk variables (*p* < 0.05). In comparison to the conventional TNM staging model, the Cox proportional risk regression model exhibited a higher C-index. After controlling for competing risk events, age, N stage, presence of perineural infiltration, number of tumor deposits, number of regional lymph nodes examined, and number of positive regional lymph nodes were independent predictors of the risk of cancer-specific mortality (*p* < 0.05).

**Conclusion:**

We have developed a prognostic model to predict the survival of patients with synchronous CRLM who undergo preoperative chemotherapy and surgery. This model has been tested internally and externally, confirming its accuracy and reliability.

**Supplementary Information:**

The online version contains supplementary material available at 10.1007/s12094-024-03513-5.

## Introduction

According to Global Cancer Statistics, colorectal cancer (CRC) ranks third in terms of incidence rate and second in terms of fatality rate among malignant tumors. According to the American Cancer Society, the 5-year survival rate for colorectal cancer with distant metastases is only 14%. Approximately 26.5% of these can be ascribed to liver metastases of colorectal cancer (CRLM) [1]. The median overall survival (mOS) of CRLM patients was only 6.9 months, whereas CRLM patients who underwent surgical resection of liver metastases had a much longer median survival of up to 35 months [2–4]. Due to the substantial impact of surgery on the outlook for patients, the treatment approach focuses on enhancing the availability of surgical operations to a broader patient population. Neoadjuvant therapy is recommended for CRLM patients that can be entirely removed during the initial surgery (R0 resected) to enhance their chances of long-term survival without tumors [5]. Nevertheless, a significant proportion of patients (up to 70%) diagnosed with CRLM are deemed inoperable at the time of initial diagnosis [6]. In such cases, it is crucial to promptly and vigorously administer comprehensive treatment to minimize the tumor burden and potentially qualify for surgical intervention, also known as conversion therapy.

Presently, the rates of response for chemotherapy along with targeted therapy can be elevated to 60% in patients with initially unresectable CRLM [7, 8]. The assessment of whether liver metastases can be surgically removed is made by a multidisciplinary team (MDT). However, there is a lack of defined criteria to determine whether patients with initially inoperable CRLM should undergo palliative or translational therapy. Hence, it is crucial to establish a scoring system that evaluates the survival advantage of patients with CRLM following conversion or neoadjuvant chemotherapy in conjunction with surgical resection. A range of predictive systems have been created to determine whether patients will derive advantages from undergoing surgery. One of the most frequently cited is the Clinical Risk Score (CRS), which predicts a patient’s overall survival (OS) based on five variables [9]. Due to its reliance on a solitary facility and a restricted number of patients, the external validity and relevance of this study are subject to debate among various population groups [10, 11]. Other well established models include those of Nordlinger [12], Iwatsuki [13], the Genetic And Morphological Evaluation (GAME) score [14], and the modified clinical score (m-CS) [15] have been adapted to genomic and chemotherapeutic variables by including KRAS mutation status in the modern era. Nevertheless, there is still ongoing debate over the influence of some variables on survival outcomes, and there is a dearth of accurate prognostic assessment for CRLM patients with treatment.

We obtained data on patients pathologically diagnosed with CRC from 2000 to 2019 from the Surveillance, Epidemiology, and End Results Database (SEER). We also collected data from the Affiliated Hospital of Qingdao University from January 1, 2010, to June 1, 2022. Prognostic models were created for CRLM patients following preoperative chemotherapy and surgical treatment using Cox proportional hazards regression and competing risk regression analyses.

## Methods and materials

### Case information collection and data collation

We downloaded data of CRLM patients from the SEER database (www.seer.cancer.gov) “Incidence SEER Research Plus Data, 17 Registries, Nov 2021 Sub (2000–2019)” dataset. This database contains retrospective study information on cancer epidemiology and demographics for approximately 35% of the United States of America population. Since the confidential patient data were not included in the SEER database, ethical clearance was not necessary for the utilization of the dataset. We also collected data form the Affiliated Hospital of Qingdao University from January 1, 2010, to June 1, 2022. The study protocol was approved by the Medical Ethics Committee of the Affiliated Hospital of Qingdao University (Approval No. QDFY WZLL 28552).

### Inclusion and exclusion criteria

Filter the SEER data by the following fields: (i) {Site recode ICD-O-3/WHO 2008} = ‘Colon and Rectum’; (ii) {Behavior code ICD-O-3} = ‘Malignant’; (iii) {Chemotherapy recode (yes, no/unk)} = ‘Yes’; (iv) {RX Summ—Systemic/Sur Seq} = ‘Systemic therapy before surgery’; (v) {SEER Combined Mets at DX-liver (2010 +)} = ‘Yes’; (vi) {Diagnostic Confirmation} = ‘Positive exfoliative cytology, no positive histology’, ‘Positive histology’, ‘Positive microscopic confirm, method not specified’; (vii) {Histologic Type ICD-O-3} = ‘8140’, ‘8210’, ‘8220’, ‘8480’, ‘8481’; (viii) {RX Summ–Surg Oth Reg/Dis (2003 +)} = ‘Non-primary surgical procedure to distant site’; (ix) {Mets at DX-Other(2016 +)} = ‘None; no other metastases’; (x) {RX SUMM—SURG PRIM SITE (1998 +)} = ‘Resection’, ‘A surgical procedure to the primary site was done’.

Inclusion criteria: (i) primary site is colon, junction of colon and rectum or rectum; (ii) adenocarcinoma confirmed by microscopic pathology; (iii) liver metastases are identified either concurrently with the diagnosis of CRC or within 6 months following the surgery of primary cancer; (iv) surgical resection (R0 resection) of the primary site and liver metastasis; (v) receiving complete chemotherapy before surgery.

Exclusion criteria: (i) confirmation of multiple primary tumors; (ii) accompanied by distant metastasis to other organs and/or distant lymph node metastasis; (iii) those whose survival status is death, but the cause is unknown; (iv) those with missing follow-up dates.

The SEER data obtained after inclusion and exclusion criteria were randomly divided 1:1 into training cohort and internal validation cohort using the R (v.4.3.1) ‘splithalfr’ package. The screened data from the Affiliated Hospital of Qingdao University were defined as the external validation cohort (QDU cohort).

### Study variables and outcomes

We selected the following variables for further analysis: age, gender, marital status, race, primary tumor site, pre-treatment CEA level, tumor size, T stage, N stage, pathological grade, number of tumor deposits, perineural infiltration, number of regional lymph nodes examined, and number of positive regional lymph nodes. Among them, age, tumor size, number of tumor deposits number of regional lymph nodes examined, and number of positive regional lymph nodes were continuous variables, and the other variables were categorical variables. We transformed continuous variables into categorical variables and defined the number of categories and the choice of node locations for categorical variables by drawing Restricted Cubic Spline (RCS) analysis. OS was used as the study outcome in this study. Kaplan–Meier (K–M) survival analyses were performed within different subgroups of the SEER and QUD cohorts, respectively, and mOS was obtained by SPSS software (v.26).

### Cox proportional risk regression model and competitive risk regression model construction

Univariate Cox regression analysis was performed in the training cohort. Variables with *p* < 0.05 were considered significant for univariate Cox regression. A stepwise regression method based on Akaike information criterion (AIC) was applied to further screen the variables, and the combination of variables with the smallest AIC value was selected to be used in constructing the Cox regression model with the highest fitness. The Cox regression proportional hazards assumption test was used to test whether the model assumed that the hazard ratio (HR) changed over time.

Considering the presence of competing events of non-cancer-related factors leading to death, we further developed a competing risk regression model. Univariate analysis was performed to estimate the cumulative incidence of recurrence by cumulative incidence function (CIF). Survival curves were plotted using Nelson–Aalen cumulative risk curves. Intergroup variability was tested by Gray’s test.

### Nomogram construction and model testing

We constructed nomograms for the Cox proportional risk regression model and the competing risk regression model, respectively. Prognostic assessment of selected patients in the SEER database was performed to compare the differences in the predicted outcomes of the two nomograms. Internal validation and external validation were performed to further test the reliability and accuracy of the nomogram. Calibration curves were used to make predictions about the likelihood of outcomes occurring. The accuracy of the model was tested using the receiver operating characteristic curve (ROC) and area under curve (AUC). Decision curve analysis (DCA) was used to evaluate the clinical utility of the model.

### Statistical analyses

The study utilized R software version 4.3.1, with the major R packages utilized as follows:

(i) The ‘ggrcs’ package was utilized for conducting RCS, while ‘car’ package was utilized for analyzing the baseline patient data;
(ii) The ‘splithalfr’ package was utilized to randomly division;
(iii) The ‘survival’, ‘survminer’, and ‘coin’ packages were utilized to construct the Cox proportional risk regression models;
(iv) The ‘riskRegression’ package was utilized to construct the competitive risk models;
(v) The ‘survival’, ‘regplot’, ‘vioplot’, ‘beanplot’, ‘survivalROC’, and ‘ggDCA’ packages were utilized to create nomograms and validation;

The statistical analysis of survival analysis was performed using SPSS (v.26).

A two-sided *p* < 0.05 indicates a statistically significant difference.

## Results

### Baseline characteristics

**Fig. 1:**
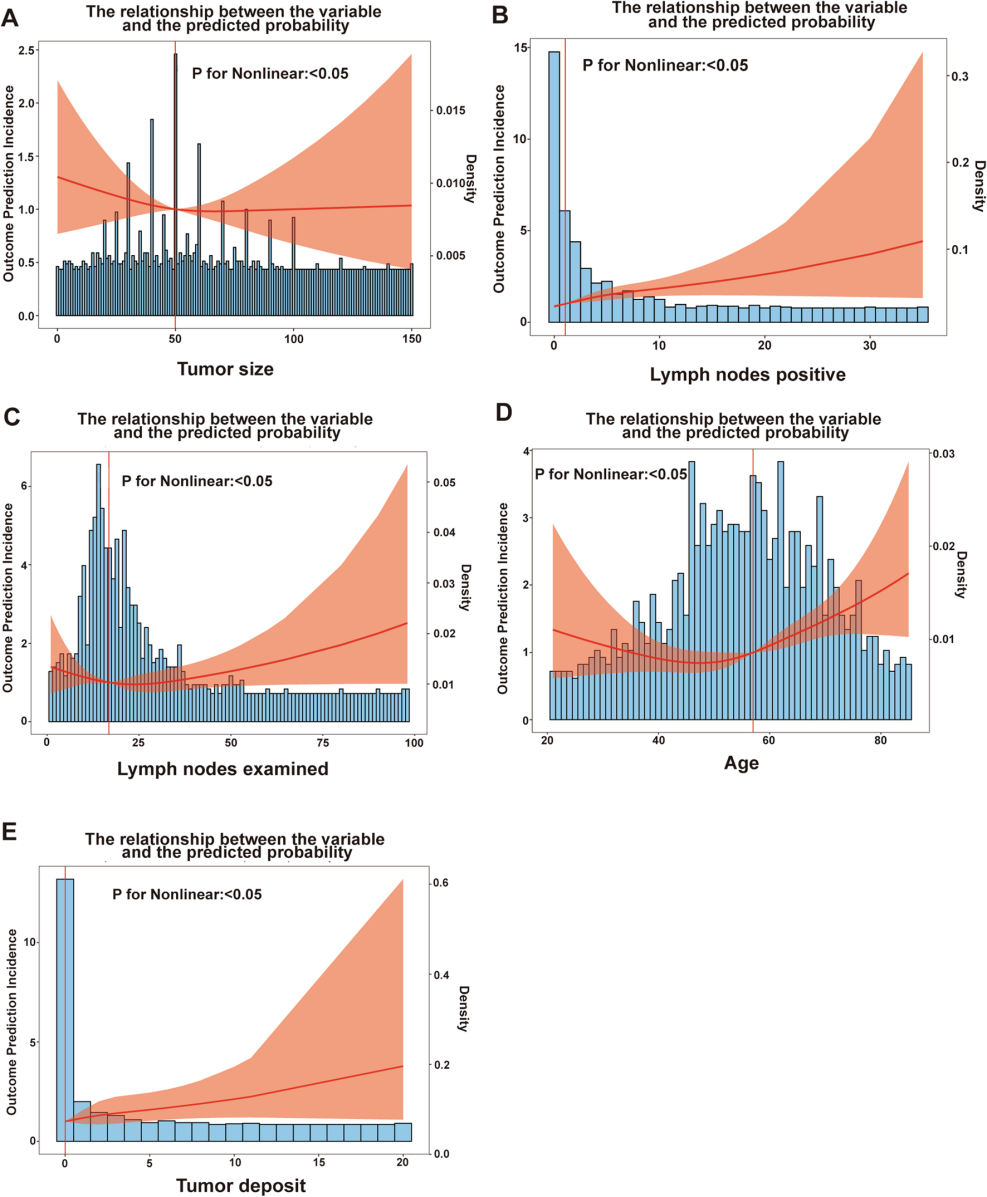
Optimal grouping nodes for continuous variables identified by RCS. **A** The best grouping node for tumor size was 49.899497 mm; **B** the best grouping node for the number of positive regional lymph nodes was 0.9657; **C** the best grouping node for the number of regional lymph nodes examined was 16.824121; **D** the best grouping node for age was 57.03015 years; and **E** the best grouping node for tumor deposits was 0.000

A total of 735 patients met the inclusion and exclusion criteria and were included in this study. Of these, 316 were in the SEER training cohort, 317 in the SEER internal validation cohort, and 102 in the external validation cohort. RCS was utilized to determine the optimal grouping nodes for the following continuous variables: age (≤ 57, > 57), tumor size (< 5 cm, ≥ 5 cm), number of tumor deposits (negative, positive), number of regional lymph nodes examined (< 17, ≥ 17), and number of regional lymph node positives (negative, positive) (Fig. 1A–E).

The baseline characteristics of all patients included in the study are shown in Tables 1 and 2. We listed the year of diagnosis of patients in the SEER and QDU cohort (Supplemental Table 1). Within the SEER cohort, the majority of individuals were male (61.5%), with Caucasians being the most prevalent ethnic group (79.9%). The primary tumor type observed most frequently was a moderately differentiated adenocarcinoma (66.0%), primarily situated in the colon (49.3%), and T3 (55.1%) and N1 (49.1%) stages being the most common. Four hundred five (64.0%) patients were accompanied by pre-treatment elevated levels of CEA. Positive regional lymph node metastases was found in 402 (63.5%) patients. One hundred forty-nine (23.5%) patients showed positive perineural infiltration, and 95 (15.0%) patients were with ≥ 1 tumor deposits. Two hundred sixteen patients (34.1%) had received radiotherapy. There were no statistically significant differences between the training cohort and the internal validation cohort in variables other than primary tumor site and pretreatment CEA level.

**Table 1:** Baseline characteristics of patients in the total SEER population, modeling cohort, and internal validation cohort

	Total SEER (n = 633)	SEER internal validation cohort (n = 317)	SEER modeling cohort (n = 316)	*p* value
Gender				0.782
Female	244 (38.5%)	120 (37.9%)	124 (39.2%)	
Male	389 (61.5%)	197 (62.1%)	192 (60.8%)	
Age				0.248
> 57	302 (47.7%)	159 (50.2%)	143 (45.3%)	
≤ 57	331 (52.3%)	158 (49.8%)	173 (54.7%)	
Race				0.803
White	506 (79.9%)	252 (79.5%)	254 (80.4%)	
Black	58 (9.2%)	28 (8.8%)	30 (9.5%)	
Others	69 (10.9%)	37 (11.7%)	32 (10.1%)	
Marital status				0.633
Married	400 (63.2%)	196 (61.8%)	204 (64.6%)	
Unmarried	211 (33.3%)	111 (35.0%)	100 (31.6%)	
Unknown	22 (3.5%)	10 (3.15%)	12 (3.8%)	
Site				0.029
Colon	312 (49.3%)	173 (54.6%)	139 (44.0%)	
Rectosigmoid junction	90 (14.2%)	40 (12.6%)	50 (15.8%)	
Rectum	231 (36.5%)	104 (32.8%)	127 (40.2%)	
Grade				0.669
Well differentiated	34 (5.4%)	18 (5.7%)	16 (5.0%)	
Moderately differentiated	418 (66.0%)	202 (63.7%)	216 (68.4%)	
Poorly differentiated	60 (9.5%)	35 (11.0%)	25 (7.9%)	
Undifferentiated	6 (1.0%)	3 (1.0%)	3 (1.0%)	
Unknown	115 (18.2%)	59 (18.6%)	56 (17.7%)	
T stage				0.699
T1	29 (4.6%)	15 (4.7%)	14 (4.3%)	
T2	33 (5.2%)	18 (5.7%)	15 (4.8%)	
T3	349 (55.1%)	171 (53.9%)	178 (56.3%)	
T4	87 (13.7%)	49 (15.5%)	38 (12.0%)	
TX	135 (21.3%)	64 (20.2%)	71 (22.5%)	
N stage				0.898
N0	182 (28.8%)	92 (29.0%)	90 (28.5%)	
N1	311 (49.1%)	153 (48.3%)	158 (50.0%)	
N2	140 (22.1%)	72 (22.7%)	68 (21.5%)	
CEA				0.011
Elevated	405 (64.0%)	187 (59.0%)	218 (69.0%)	
Normal	228 (36.0%)	130 (41.0%)	98 (31.0%)	
Perineural invasion				0.732
Negative	396 (62.6%)	203 (64.0%)	193 (61.1%)	
Positive	149 (23.5%)	71 (22.4%)	78 (24.7%)	
Unknown	88 (13.9%)	43 (13.6%)	45 (14.2%)	
Deposit				0.745
Negative	387 (61.1%)	193 (60.9%)	194 (61.4%)	
Positive	95 (15.0%)	45 (14.2%)	50 (15.8%)	
Unknown	151 (23.9%)	79 (24.9%)	72 (22.8%)	
Tumor size				0.808
< 5 cm	233 (36.8%)	114 (36.0%)	119 (37.7%)	
≥ 5 cm	245 (38.7%)	122 (38.5%)	123 (38.9%)	
Unknown	155 (24.5%)	81 (25.6%)	74 (23.4%)	
Lymph nodes examined				0.347
< 17	265 (41.9%)	141 (44.5%)	124 (39.2%)	
≥ 17	328 (51.8%)	158 (49.8%)	170 (53.8%)	
None	35 (5.5%)	17 (5.4%)	18 (5.7%)	
Unknown	5 (0.8%)	1 (0.3%)	4 (1.3%)	
Lymph nodes positive				0.528
Negative	231 (36.5%)	120 (37.9%)	111 (35.1%)	
Positive	402 (63.5%)	197 (62.1%)	205 (64.9%)	
Sequence of surgery and radiotherapy				0.155
Surgery only	417 (65.9%)	211 (66.6%)	206 (65.2%)	
Post-surgery	32 (5.1%)	19 (6.00%)	13 (4.1%)	
Both	9 (1.4%)	7 (2.2%)	2 (0.6%)	
Pre-surgery	175 (27.6%)	80 (25.2%)	95 (30.1%)	
Radiotherapy type				0.935
Unknown/none	417 (65.9%)	211 (66.6%)	206 (65.2%)	
Beam radiation	208 (32.9%)	102 (32.2%)	106 (33.5%)	
Implant or radioisotope	8 (1.3%)	4 (1.2%)	4 (1.3%)

**Table 2:** Baseline characteristics of patients in the total population, SEER cohort, and QDU external validation cohort

	Total (n = 735)	SEER cohort (n = 633)	QDU external validation cohort (n = 102)	*p* value
Gender				0.005
Female	268 (36.5%)	244 (38.5%)	24 (23.5%)	
Male	467 (63.5%)	389 (61.5%)	78 (76.5%)	
Age				< 0.001
> 57	376 (51.2%)	302 (47.7%)	74 (72.5%)	
≤ 57	359 (48.8%)	331 (52.3%)	28 (27.5%)	
Marital status				< 0.001
Married	502 (68.3%)	400 (63.2%)	102 (100%)	
Unmarried	22 (3.0%)	211 (33.3%)	0 (0%)	
Unknown	211 (28.7%)	22 (3.48%)	0 (0%)	
Site				< 0.001
Colon	361 (49.1%)	312 (49.3%)	49 (48.0%)	
Rectosigmoid junction	90 (12.2%)	90 (14.2%)	0 (0%)	
Rectum	284 (38.6%)	231 (36.5%)	53 (52.2%)	
Grade				< 0.001
Well differentiated	40 (5.4%)	34 (5.4%)	6 (5.9%)	
Moderately differentiated	499 (67.9%)	418 (66.0%)	81 (79.4%)	
Poorly differentiated	75 (10.2%)	60 (9.5%)	15 (14.7%)	
Undifferentiated	6 (0.8%)	6 (1.0%)	0 (0%)	
Unknown	115 (15.6%)	115 (18.2%)	0 (0%)	
T stage				< 0.001
T1	29 (4.0%)	29 (4.6%)	0 (0%)	
T2	45 (6.1%)	33 (5.21%)	12 (11.8%)	
T3	398 (54.1%)	349 (55.1%)	49 (48.0%)	
T4	122 (16.6%)	87 (13.7%)	35 (34.3%)	
TX	141 (19.2%)	135 (21.3%)	6 (5.9%)	
N stage				0.001
N0	217 (29.5%)	182 (28.8%)	35 (34.3%)	
N1	342 (46.5%)	311 (49.1%)	31 (30.4%)	
N2	176 (23.9%)	140 (22.1%)	36 (35.3%)	
CEA				0.679
Elevated	473 (64.4%)	405 (64.0%)	68 (66.7%)	
Normal	262 (35.6%)	228 (36.0%)	34 (33.3%)	
Perineural invasion				< 0.001
Negative	438 (59.6%)	396 (62.6%)	42 (41.2%)	
Positive	188 (25.6%)	149 (23.5%)	39 (38.2%)	
Unknown	109 (14.8%)	88 (13.9%)	21 (20.6%)	
Deposit				0.195
Negative	452 (61.5%)	387 (61.1%)	65 (63.7%)	
Positive	115 (15.6%)	95 (15.0%)	20 (19.6%)	
Unknown	168 (22.9%)	151 (23.9%)	17 (16.7%)	
Tumor size				< 0.001
< 5 cm	289 (39.3%)	233 (36.8%)	56 (54.9%)	
≥ 5 cm	282 (38.4%)	245 (38.7%)	37 (36.3%)	
Unknown	164 (22.3%)	155 (24.5%)	9 (8.8%)	
Lymph nodes examined				< 0.001
< 17	330 (44.9%)	265 (41.9%)	65 (63.7%)	
≥ 17	364 (49.5%)	328 (51.8%)	36 (35.3%)	
None	35 (4.8%)	35 (5.53%)	0 (0%)	
Unknown	6 (0.8%)	5 (0.8%)	1 (1.0%)	
Lymph nodes positive				0.754
Negative	266 (36.2%)	231 (36.5%)	35 (34.3%)	
Positive	469 (63.8%)	402 (63.5%)	67 (65.7%)	
Sequence of surgery and radiotherapy				< 0.001
Surgery only	508 (69.1%)	417 (65.9%)	80 (78.4%)	
Post-surgery	41 (5.6%)	32 (5.1%)	12 (11.8%)	
Both	9 (1.2%)	9 (1.4%)	2 (2.0%)	
Pre-surgery	177 (24.1%)	175 (27.6%)	6 (5.8%)	
Radiotherapy type				0.011
Unknown/None	508 (69.1%)	417 (65.9%)	82 (78.4%)	
Beam radiation	219 (29.8%)	208 (32.9%)	20 (19.6%)	
Implant or Radioisotope	8 (1.1%)	8 (1.3%)	0 (0.0%)	

Baseline characteristics of the external validation cohort revealed that a significant majority of the patients were male (76.5%) and over the age of 57 (72.5%). 52.0% patients had a primary tumor in the rectum, and 79.4% patients were moderately differentiated adenocarcinoma. 63.7% patients had negative tumor deposits and 65.7% patients had positive regional lymph node metastases. There were 20 patients (19.6%) in the external validation cohort who had received radiotherapy (Table 2).

### Results of overall and subgroup survival analyses

**Fig. 2:**
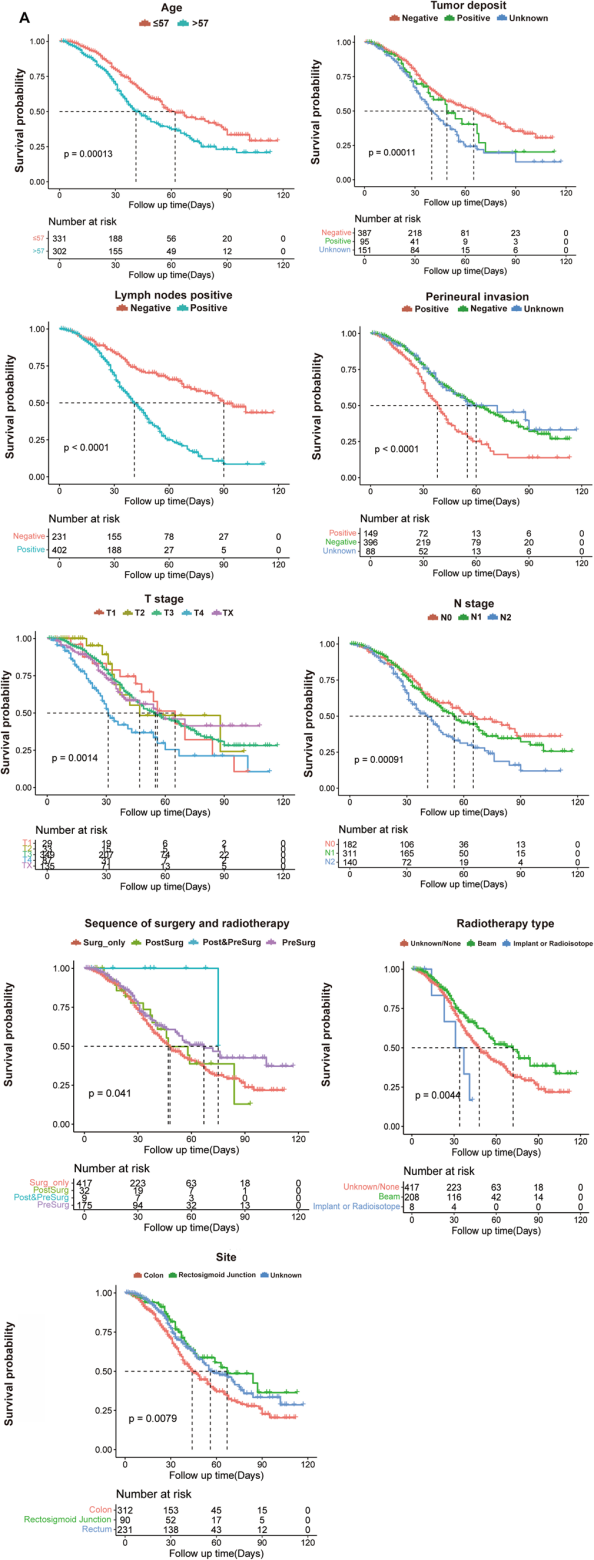

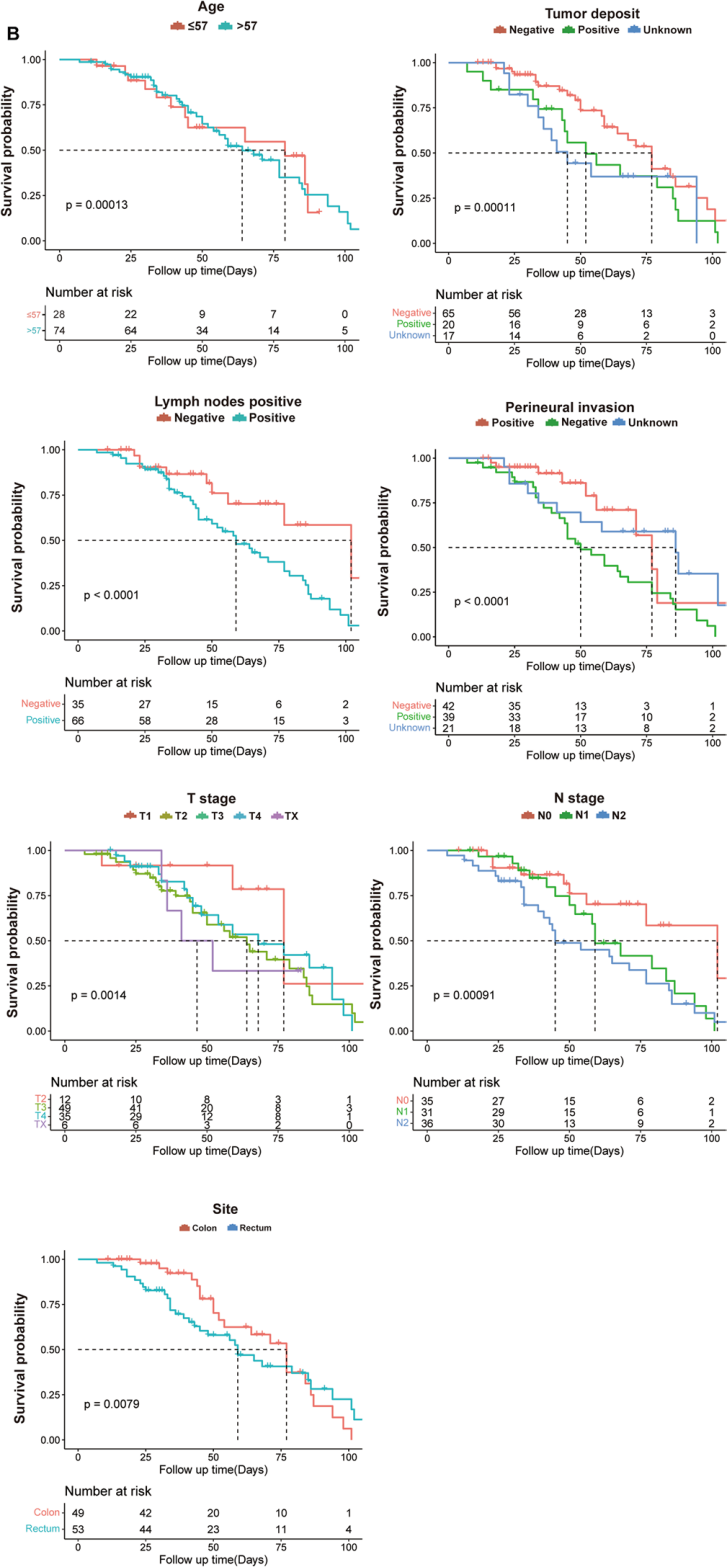
Kaplan–Meier survival analysis at SEER cohort and QDU external validation cohort. **A** The SEER cohort showed significant survival differences in age, number of tumor deposits, number of positive regional lymph nodes, perineural infiltration, T stage, N stage and tumor site subgroups (*p* < 0.05); **B** the QDU cohort showed significant survival differences (*p* < 0.05)

The mean survival time of the training cohort was 64.24 months (95% CI 58.50–69.97) with a mOS of 55.00 months (95% CI 46.97–63.03), the mOS of the internal validation cohort was 60.27 months (95% CI 54.91–65.63) with a mOS of 48.00 months (95% CI 40.65–55.35), and the external validation cohort had a mean survival time of 65.84 months (95% CI 59.34–72.34) and a mOS of 68.00 months (95% CI 54.91–81.08). Subgroup K–M analysis of the SEER cohort showed significant survival differences (*p* < 0.05) in age, number of tumor deposits, number of positive regional lymph nodes, presence of perineural infiltration, subgroups of T stage, N stage, and primary tumor site (Fig. 2A). Subgroup K–M survival analysis of the external validation cohort similarly yielded significant survival differences (*p* < 0.05) in subgroups of the above variables (Fig. 2B). The mOS of all subgroups is shown in Table 3.

**Table 3:** mOS of SEER cohort and QDU external validation

	SEER cohort	QDU external validation
	mOS ± SB (95%CI)	mOS ± SB (95%CI)
*Gender*		
Female	49.000 ± 5.258 (38.694–59.306)	68.000 ± 12.923 (42.672–93.328)
Male	54.000 ± 3.505 (47.131–60.869)	65.000 ± 6.508 (52.243–77.756)
*Age*		
> 57	41.000 ± 3.059 (35.004–46.996)	64.000 ± 7.658 (48.990–79.010)
≤ 57	62.000 ± 5.798 (50.636–73.364)	79.000 ± 16.470 (46.719–111.281)
*Race*		
White	68.000 ± 21.997 (24.887–111.113)	NA
Black	70.000 ± 8.931 (52.495–87.505)	NA
Others	49.000 ± 3.089 (42.946–55.054)	NA
*Marital status*		
Married	56.000 ± 4.977 (46.244–65.756)	NA
Unmarried	47.000 ± 4.336 (38.502–55.498)	NA
Unknown	33.000 ± 10.708 (12.012–53.988)	NA
*Site*		
Colon	44.000 ± 3.135 (37.855–50.145)	77.000 ± 6.453 (64.351–89.649)
Rectosigmoid Junction	67.000 ± 14.661 (38.264–95.736)	NA
Rectum	56.000 ± 7.391 (41.513–70.487)	59.000 ± 6.070 (47.104–70.896)
*Grade*		
Well differentiated	58.000 ± NA (NA)	NA
Moderately differentiated	52.000 ± 3.377 (45.382–58.618)	NA
Poorly differentiated	41.000 ± 3.079 (34.965–47.035)	NA
Undifferentiated	24.000 ± 20.821 (0.000–64.808)	NA
Unknown	65.000 ± 8.218 (48.892–81.108)	NA
*T stage*		
T1	65.000 ± 9.116 (47.132–82.868)	NA
T2	47.000 ± 19.142 (9.481–84.519)	77.000 ± 7.519 (62.262–91.738)
T3	55.000 ± 4.840 (45.514–64.486)	64.000 ± 6.914 (50.448–77.552)
T4	31.000 ± 2.724 (25.661–36.339)	68.000 ± 15.041 (38.520–97.480)
TX	56.000 ± 10.174 (36.060–75.940)	41.000 ± 9.798 (21.796–60.204)
*N stage*		
N0	65.000 ± 10.854 (43.726–86.2744)	102.000 ± 18.599 (65.547–138.453)
N1	55.000 ± 5.043 (45.116–64.884)	59.000 ± 6.299 (46.654–71.346)
N2	41.000 ± 3.854 (33.446–48.554)	45.000 ± 12.586 (20.332–69.668)
*CEA*		
Elevated	49.000 ± 3.535 (42.071–55.929)	65.000 ± 6.042 (53.158–76.842)
Normal	67.000 ± 8.701 (49.946–84.054)	77.000 ± 22.615 (32.675–121.325)
*Perineural invasion*		
Negative	60.000 ± 4.815 (50.563–69.437)	77.000 ± 5.900 (65.435–88.565)
Positive	38.000 ± 3.122 (31.880–44.120)	50.000 ± 5.345 (39.524–60.476)
Unknown	55.000 ± 16.584 (47.041–58.959)	86.000 ± 17.043 (52.596–119.404)
*Deposit*		
Negative	65.000 ± 6.598 (52.067–77.933)	77.000 ± 5.136 (66.933–87.067)
Positive	49.000 ± 7.153 (34.979–63.021)	52.000 ± 10.725 (30.978–73.022)
Unknown	40.000 ± 2.951 (34.217–45.783)	45.000 ± 5.885 (33.465–56.535)
*Tumor size*		
< 5 cm	47.000 ± 3.645 (39.856–54.144)	59.000 ± 11.706 (36.056–81.944)
≥ 5 cm	67.000 ± 7.939 (51.439–82.561)	79.000 ± 12.639 (54.228–103.772)
Unknown	51.000 ± 5.846 (39.541–62.459)	56.000 ± 16.973 (22.733–89.267)
*Lymph nodes examined*		
< 17	49.000 ± 4.196 (40.776–57.224)	59.000 ± 8.088 (43.147–74.853)
≥ 17	55.000 ± 5.747 (43.735–66.265)	77.000 ± 12.900 (51.716–102.284)
None	56.000 ± 11.998 (32.483–79.517)	
Unknown	51.000 ± 37.245 (0.000–124.000)	41.000 ± NA (NA)
*Lymph nodes positive*		
Negative	90.000 ± 8.441 (73.456–106.544)	102.000 ± 18.599 (65.547–138.453)
Positive	41.000 ± 2.275 (36.541–45.459)	59.000 ± 6.732 (45.805–72.195)

### Cox proportional risk regression model and nomogram

**Fig. 3:**
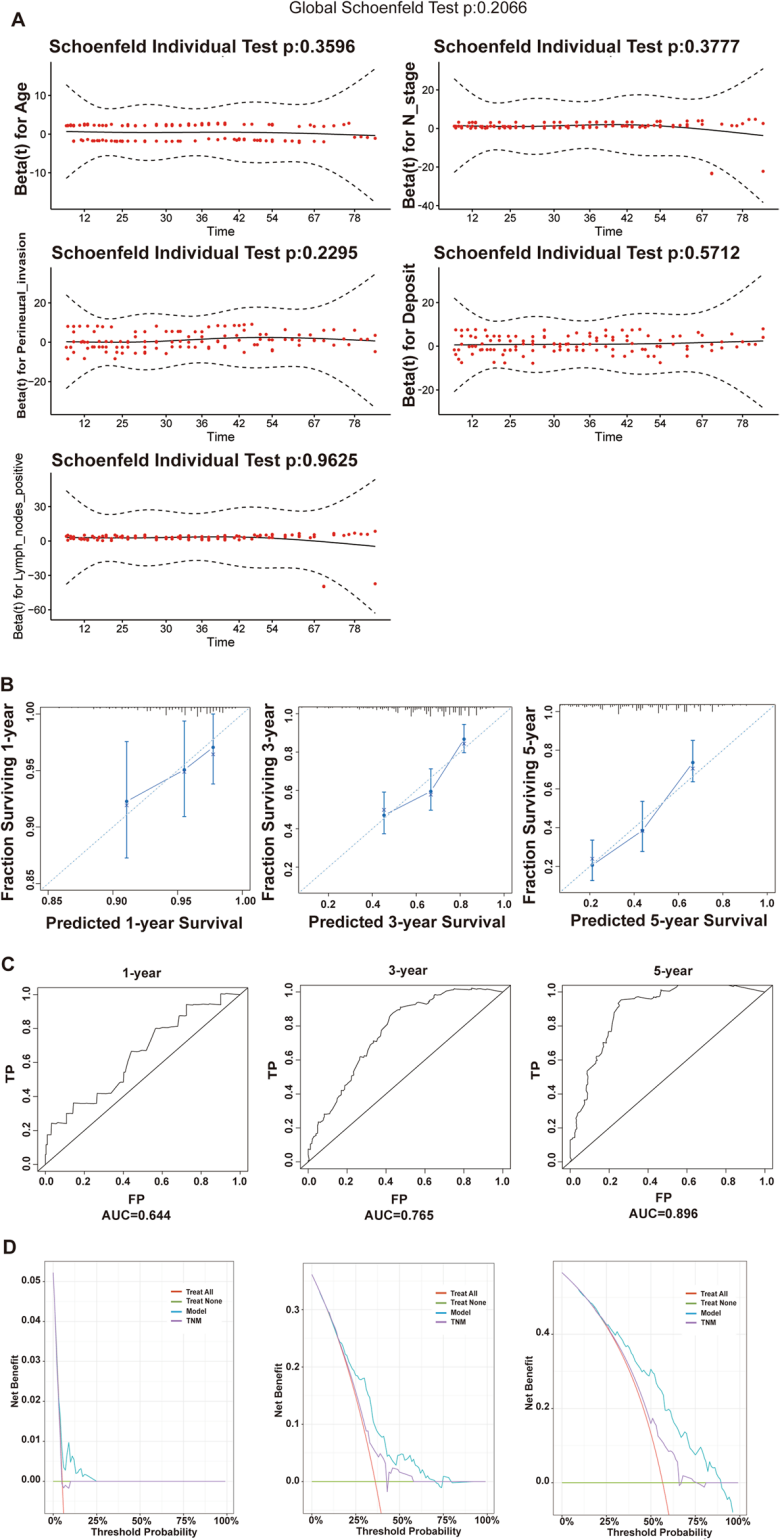

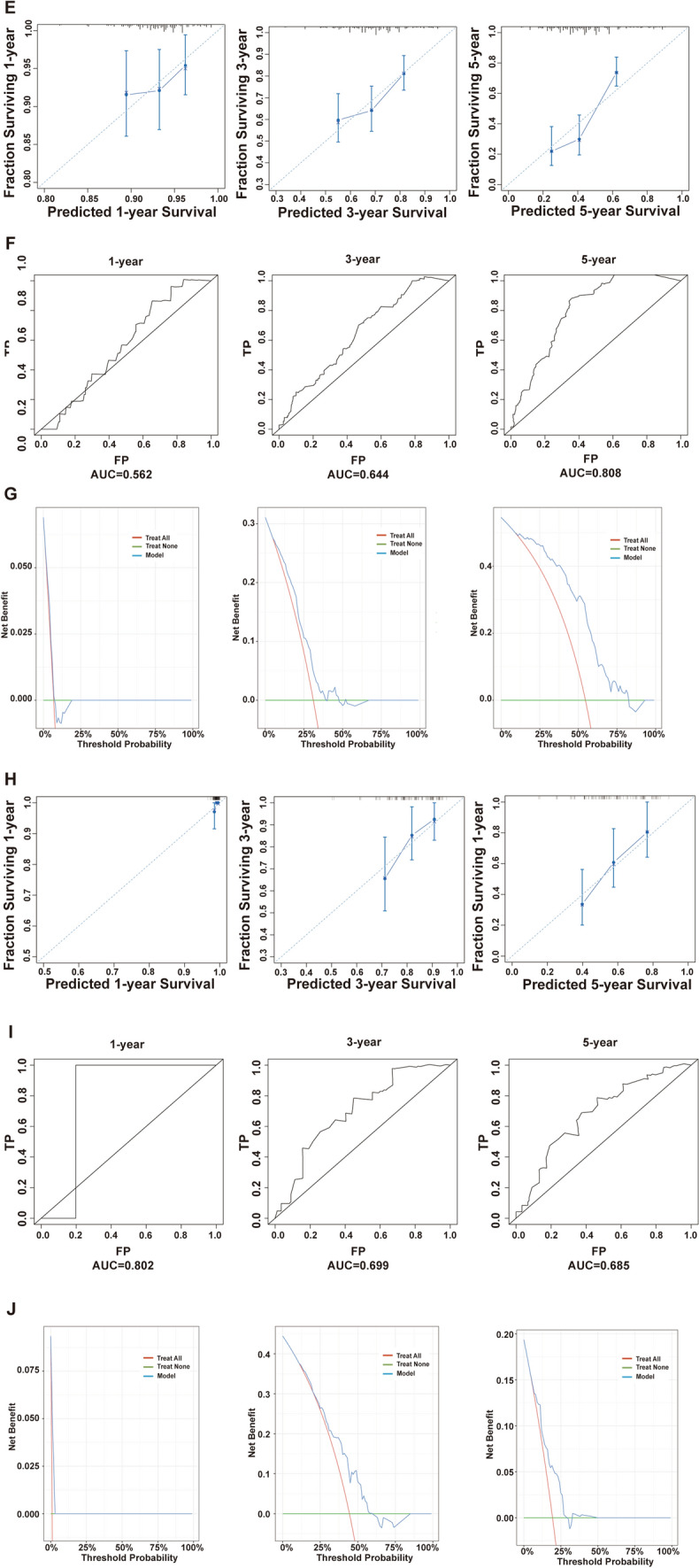
Cox proportional risk regression modeling and testing. **A** Age, N stage, number of tumor deposits, number of positive regional lymph nodes and perineural infiltration met the Cox regression proportional risk assumption test (*p* > 0.05); **B** calibration curves of the modeling set at 1 year, 3 years, and 5 years, respectively; **C** ROC curves of the modeling set at 1 year, 3 years, and 5 years, respectively, and the corresponding AUC values; **D** DCA curves of the modeling set at 1 year, 3 years, and 5 years, respectively, and compared with the TNM prediction model; **E** calibration curves for the internal validation set at 1 year, 3 years, and 5 years, respectively; **F** ROC curves and corresponding AUC values for the internal validation set at 1 year, 3 years, and 5 years, respectively; **G** DCA curves for the internal validation set at 1 year, 3 years, and 5 years, respectively; **H** calibration curves for the external validation set at 1 year, 3 years, and 5 years, respectively; **I** external ROC curves and corresponding AUC values for the validation set at 1 year, 3 years, and 5 years, respectively; **J** DCA curves for the external validation set at 1 year, 3 years, and 5 years, respectively. Model: the Cox proportional risk regression model, and TNM: the TNM prediction model

Univariate Cox regression analysis was performed on the training cohort. Age, T stage, N stage, presence of perineural infiltration, number of tumor deposits, number of positive regional lymph nodes and tumor size were verified as significant variables (*p* < 0.05) (Table 4). Variables were screened for the training cohort by AIC stepwise regression method and included in multivariate Cox regression analysis. Finally, age, N stage, presence of perineural infiltration, number of tumor deposits and number of positive regional lymph nodes were obtained as independent risk factors for prognosis (*p* < 0.05) (Table 4). These variables met the Cox regression proportional risk assumption test (*p* > 0.05) (Fig. 3A).

**Table 4:** Modeling cohort for univariate and multivariate Cox regression

Variables	Univariate cox			Multivariate cox		
	HR	95%CI	*p* value	HR	95%CI	*p* value
Gender			0.9			
Female						
Male	0.974	0.678–1.398				
Age			** < 0.05**			** < 0.05**
> 57						
≤ 57	1.54	1.086–2.185		1.521	1.061–2.180	
Race			0.5			
White						
Black	0.646	0.315–1.326				
Others	0.959	0.539–1.707				
Marital status			0.3			
Married						
Unmarried	1.329	0.901–1.961				
Unknown	1.120	0.398–3.153				
Site			0.8			
Colon						
Rectosigmoid junction	0.851	0.491–1.473				
Rectum	0.919	0.631–1.338				
Grade			0.07			
Well differentiated						
Moderately differentiated	2.536	0.802–8.013				
Poorly differentiated	4.4566	0.897–15.313				
Undifferentiated	1.818	0.303–10.905				
Unknown	1.782	0.519–6.119				
T stage			0.06			
T1						
T2	1.090	0.315–3.770				
T3	1.151	0.464–2.851				
T4	2.400	0.892–6.456				
TX	1.179	0.452–3.077				
N stage			**< 0.05**			** < 0.05**
N0						
N1	1.441	0.929–2.234		0.187	0.056–0.624	
N2	2.281	1.406–3.702		0.183	0.051–0.662	
CEA			0.2			
Elevated						
Normal	1.261	0.859–1.849				
Perineural invasion			** < 0.05**			** < 0.05**
Negative						
Positive	0.575	0.389–0.848		0.628	0.409–0.965	
Unknown	0.481	0.261–0.887		0.464	0.233–0.926	
Deposit			** < 0.05**			**< 0.05**
Negative						
Positive	0.954	0.536–1.697		0.695	0.366–1.319	
Unknown	1.771	1.212–2.587		1.717	1.130–2.610	
Tumor size			0.3			0.155
< 5 cm						
≥ 5 cm	1.303	0.799–2.125		1.465	0.866–2.477	
Unknown	0.990	0.593–1.655		1.026	0.901–1.752	
Lymph nodes examined			0.4			
< 17						
≥ 17	0.697	0.144–3.362				
None	1.173	0.286–4.819				
Unknown	0.914	0.222–3.758				
Lymph nodes positive			** < 0.001**			** < 0.05**
Negative						
Positive	3.208	2.128–4.836		10.965	3.348–35.913	
Sequence of surgery and radiotherapy			0.6			
Surgery only						
Pre-surgery	0.785	0.513–1.200	0.264			
Post-surgery	1.175	0.570–2.421	0.662			
Both	0.000	–	0.995			
Radiotherapy type			0.4			
None						
Beam radiation	0.821	0.553–1.218	0.327			
Implant or Radioisotope	1.747	0.429–7.121	0.436			

*P*-values that are bold indicate statistical significance

Calibration curves, ROC curves, and DCA curves were plotted in the internal validation set and the external validation set at 1 year, 3 years, and 5 years, respectively. The model’s performance and accuracy are deemed satisfactory (Fig. 3B–J). The C-index and DCA curves suggested that the Cox model had higher clinical predictive and application value than TNM staging (Table 5, Fig. 3D).

**Table 5:** C-index for Cox proportional risk regression model and competitive risk model

Model	SEER modelling cohort		SEER internal validation cohort		QDU external validation	
	C-index	95%Cl	C-index	95%Cl	C-index	95%Cl
*Cox proportional risk regression model*						
Nomogram	0.673	0.646–0.700	0.642	0.590–0.694	0.671	0.591–0.751
TNM	0.612	0.560–0.664	0.581	0.529–0.633	0.647	0.558–0.735
*Competitive risk model*						
1-year	0.623	NA	NA	NA	NA	NA
3-year	0.682	NA	NA	NA	NA	NA
5-year	0.703	NA	NA	NA	NA	NA

**Fig. 4:**
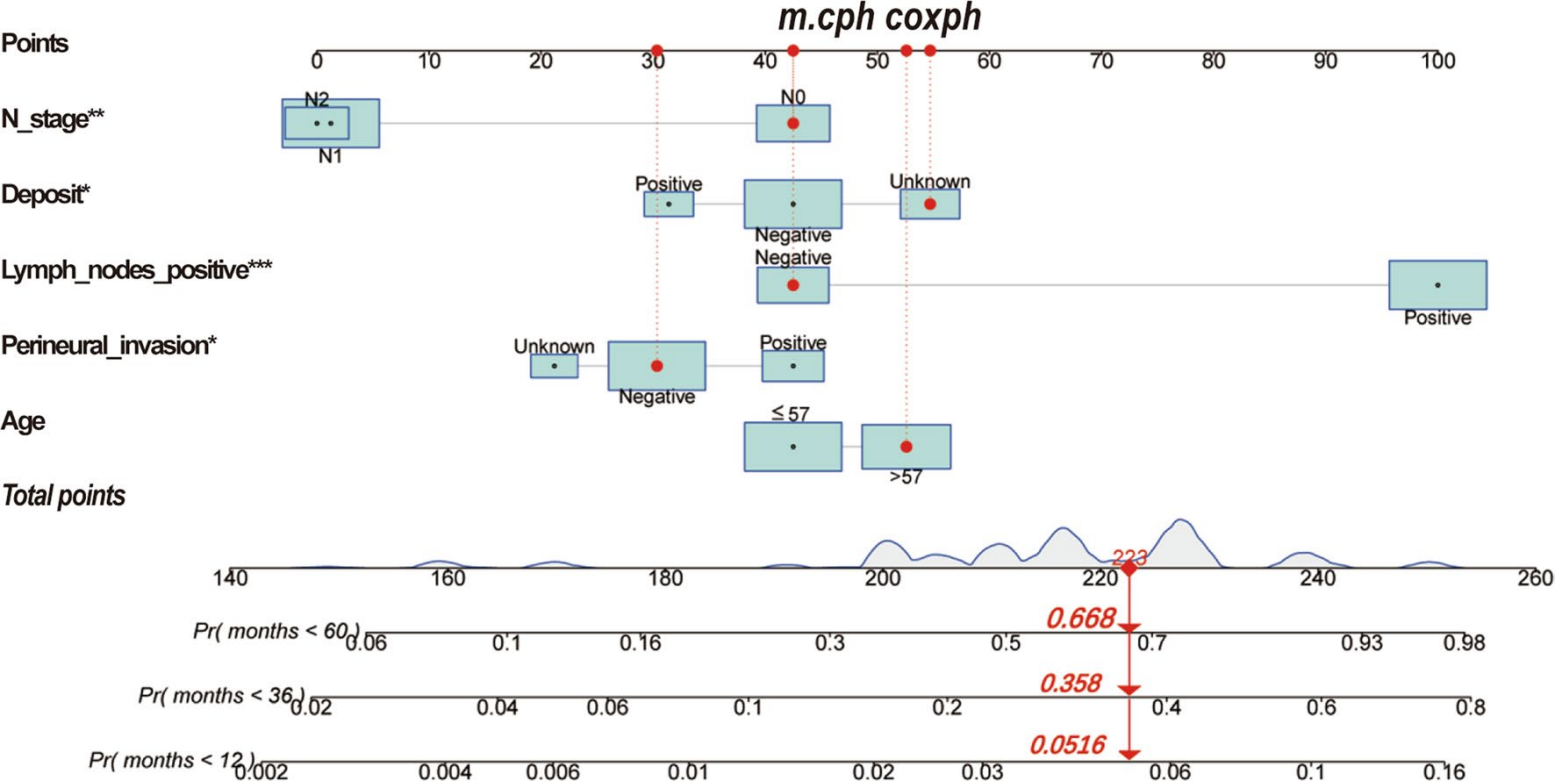
Cox proportional risk regression model nomogram

We constructed Cox proportional risk regression nomogram at 1 year, 3 years, and 5 years, respectively (Fig. 4A). The nomogram represents the covariate values for a specific patient (ID = 35,288,445) in the SEER dataset. The total points was used to calculate the probability of the patient having a survival time of less than 1, 3, and 5 years. The probabilities were found to be 0.0516, 0.358, and 0.668, respectively. (Fig. 4A).

### Competitive risk regression model and nomogram

**Fig. 5:**
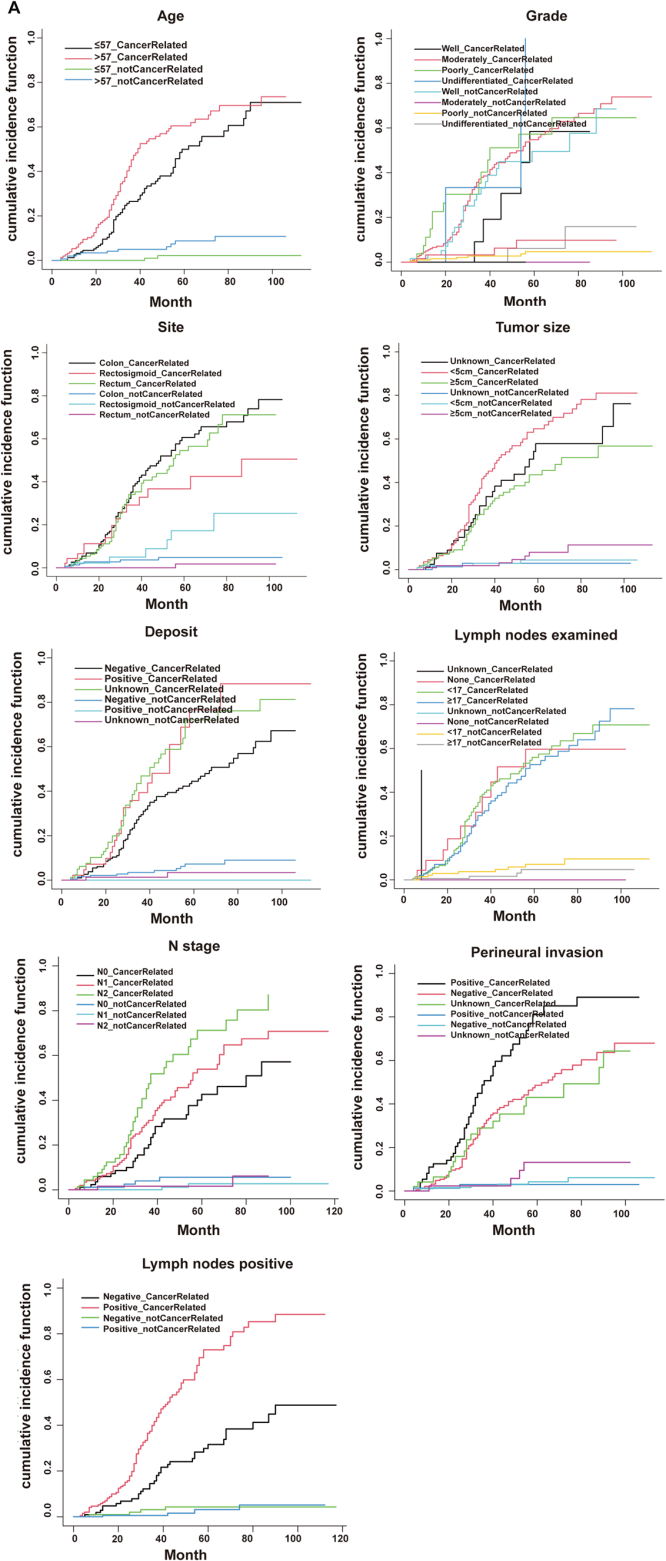

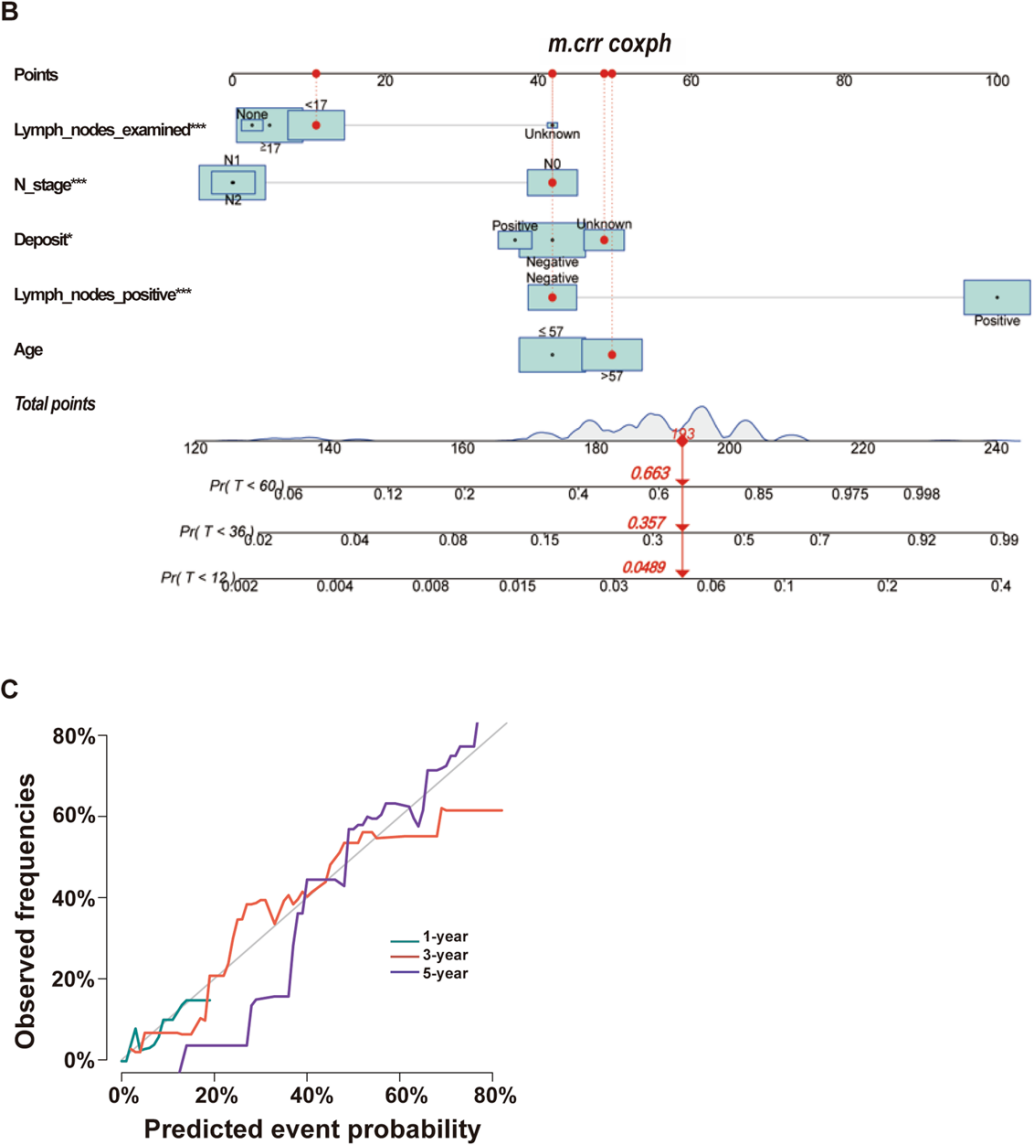
Competition risk modeling and testing. **A** Nelson–Aalen curves for the variables age, pathological stage, tumor site, tumor size, tumor deposits, number of regional lymph nodes examined, N stage, number of positive regional lymph nodes, and perineural infiltration. **B** Competing risk model nomogram. id = 35,288,445 The patient’s total score is 193, and his cumulative probability of death at 1 year, 3 years, and 5 years is predicted to be: 0.0489, 0.357 and 0.663, respectively. **C** Calibration curves for the modeling set at 1 year, 3 years, and 5 years, respectively

Gray’s test was used to test for between-group variability. CIF was used to estimate the cumulative incidence of recurrence, and Nelson–Aalen cumulative risk curves were developed. There was a statistically significant difference in the risk of death between subgroups in the variables of age, number of regional lymph nodes examined, tumor size, N stage, presence of perineural infiltration, number of tumor deposits, and number of positive regional lymph nodes after controlling for competing risk events (*p* < 0.05). There was a statistically significant difference (*p* < 0.05) in the cumulative competing risks in age and primary tumor sites (Table 6, Fig. 5A). A multivariable competing risk regression model was constructed and visualized by the nomogram (Fig. 5B). The model was further validated with C-indexes of 0.623, 0.682, and 0.703 at 1 year, 3 years, and 5 years, respectively (Table 5). The nomogram calculated the likelihood of survival for the same patient with id = 35,288,445 at less than 1, 3, and 5 years. The given probability represents the likelihood of death while taking into account other risk events, namely with probabilities of 0.0443, 0.327, and 0.629, respectively (Fig. 5B). There was a discernible disparity in the calculation of cumulative risk of death between the competing risk model and the Cox proportional risk model. The competing risk model yields a slightly lower risk of death for patients with id = 35,288,445. The calibration curve at 1 year, 3 years, and 5 years is shown in Fig. 5C.

**Table 6:** Modeling cohort for Gray’s test

Variables	Death risk of cancer		Death risk of competitive events	
	Statistic	*p* value	Statistic	*p* value
Gender	0.256	0.613	0.218	0.641
Age	6.243	**0.012**	6.758	**0.009**
Race	1.212	0.546	1.427	0.490
Marital status	1.943	0.379	2.227	0.328
Site	2.841	0.241	12.716	**0.002**
Grade	3.910	0.418	2.789	0.594
T stage	5.100	0.277	4.116	0.391
N stage	12.004	**0.002**	1.906	0.386
CEA	0.585	0.444	0.795	0.372
Perineural infiltration	17.396	**0.000**	2.341	0.310
Deposit	11.565	**0.003**	3.269	0.195
Tumor size	9.502	**0.009**	1.485	0.476
Lymph nodes examined	18.428	**0.000**	2.806	0.423
Lymph nodes positive	32.451	** < 0.001**	0.212	0.645

## Discussion

The primary benefit of preoperative chemotherapy in CRLM patients is the potential to render previously inoperable tumors operable, a concept commonly known as “conversion therapy”. Conversion therapy has the potential to decrease the stage of cancer to a resectable level in approximately 35% of cases in these patients [16]. Preoperative chemotherapy can have benefits in decreasing the stage, even in patients with originally resectable illness. Meanwhile, preoperative chemotherapy facilitates the treatment of micrometastatic lesions and enhances the ability to achieve negative margins after surgery [17]. Nevertheless, the intricate individual diversity of colorectal liver metastases (CRLM) indicates that a solitary biomarker or predictor in this group may not accurately reflect the intricate tumor characteristics of individuals with initially inoperable CRLM. The primary factors influencing survival time in this patient population are the lack of successful conversion and the recurrence of postoperative illness. The long-term consequences of conversion treatment continue to be a subject of debate and disagreement. A considerable proportion of patients encounter early relapse [16, 18]. A retrospective study showed that patients with conversion therapy combined with surgery had a mOS of 24 months, whereas patients with conversion failure had a mOS of only 14 months (*p* < 0.001), which was similar to that of patients on palliative chemotherapy [19]. This implies that it is crucial to evaluate our patients who may derive advantages from conversion therapy, as well as preoperative chemotherapy.

The determination of “unresectability” varies depending on the surgeon, the accuracy of cross-sectional imaging tests, and guidelines that have not yet been standardized. Resection of liver metastases needs to be performed to ensure complete (R0) resection of the liver metastases and to preserve sufficient functional liver tissue. Factors such as the number and location of liver metastases are no longer used as single factors in determining the feasibility of surgery [20, 21]. However, a clear distinction between initially resectable and initially unresectable lesions remains relatively difficult. From an oncological point of view, perioperative chemotherapy is recommended even if the lesion is anatomically resectable; induction chemotherapy followed by surgery is also recommended for patients with borderline resectable CRLM [22]. Therefore, our study included CRLM that was initially resectable but required preoperative neoadjuvant therapy as well as initially unresectable for conversion therapy in one patient population. We aimed to investigate the influencing factors that can affect the prognosis of patients with CRLM treated with preoperative chemotherapy combined with surgical resection.

Our model incorporated several characteristics that are supported by existing scoring systems. Preoperative CEA levels have been observed to have an impact on both overall survival (mOS) and the response to systemic therapy [9, 23], presence of positive tumor deposits in CRLM patients is also associated with a worse outcome [24]. A tumor deposit is a distinct tumor mass located in the pericolonic or perirectal fat or nearby mesentery (colonic mesenteric fat), separate from the main tumor infiltrate and without any remaining lymphoid tissue [25]. The 8th of the American Joint Committee on Cancer (AJCC) TNM staging classifies lesions that are negative regional lymph nodes and positive tumor deposits as N1c. The correlation between the existence and quantity of tumor deposits and the unfavorable postoperative prognosis in CRC is substantial. Furthermore, there is increasing endorsement for considering tumor deposits as an indicator of distant metastasis [26–28]. Perineural invasion (PNI) is one of the most potent interactions between tumors and nerves. PNI is interpreted as tumor proximity to nerves, occupying at least 33% of the nerve circumference, or the presence of tumor cells within the nerve sheaths of the nerve epineurium, nerve fascicles, or nerve endothelium [29]. Stimulating the growth of nerves within the tumor enhances the advancement of cancer, and the interaction between the tumor and nerves mutually enhances the development of the tumor. Neurons release beneficial growth factors and angiogenic signals that promote the growth of tumor cells. In addition, tumors exploit nerves as an additional pathway for distant metastasis [30, 31]. PNI has been shown to correlate with a poor prognosis in a variety of malignancies such as pancreatic adenocarcinomas, squamous cell carcinomas of the head and neck, gastric and colorectal cancers [29, 31, 32]. Specifically, PNI is strongly linked to the advancement of disease and unfavorable results in CRC, and it indicates the possibility of early spread of cancer cells to other parts of the body [32]. In our patient cohort, 25.6% of patients were positive for PNI, which is consistent with data from previous studies [33–36]. The impact of the number of positive regional lymph nodes on survival after surgery has been taken into account. Research has demonstrated a direct correlation between the number of positive regional lymph nodes and pathological grade. In addition, a higher number of positive lymph nodes increases the probability of multiple metastases and is associated with a more severe response to chemotherapy [37]. Ozawa et al. [38] demonstrated that a higher number of positive regional lymph nodes was associated with worse 5-year survival in stage IV CRC patients underwent surgery. Our investigation also discovered that the presence of positive regional lymph node metastases was a prognostically independent risk factor for patients. Several prognostic studies have included the maximum diameter of liver metastases as a variable for postoperative prognostic prediction. Furthermore, previous studies have regarded the size of liver metastasis as a separate risk factor. However, Jang et al. [39] have demonstrated that the OS of patients with 1–2 liver metastases nodules is not significantly different from that of patients with 3–8 nodules. This survival outcome may be attributed to the introduction of advanced therapeutic techniques [15].

Two prognostic models were created in this investigation. The Cox proportional hazard regression model offers the benefit of analyzing the impact of multiple variables on survival outcomes and estimating survival of patients over time. In our study, the DCA curve of Cox model is always above the TNM system, suggesting that the model’s prediction has a higher net clinical benefit and better clinical application. Given the advanced age at which CRLM patients generally get diagnosed, there may be additional factors that impact cancer-specific mortality. Therefore, the competing risk regression model is used to assess the extent to which multiple variables affect the result. This study revealed that competing events exhibited a conflicting association with cancer-related outcomes in regard to age and the tumor site. The study revealed that patients over the age of 57 made up 51.2% of the overall population. Advanced age is associated with an increased risk of treatment-related adverse outcomes, including a higher likelihood of postoperative complications and readmission rates [40]. The presence of conflicting events within subgroups of tumor sites may be linked to the likelihood of postoperative problems in various surgical modalities. A study conducted by Zenger et al. [41] demonstrated that performing radical surgery on the mid-transverse colon increased the likelihood of developing gastroparesis and intestinal blockage compared to radical surgery for CRC in other locations. This finding has implications for the prognosis of patients.

After excluding other competing events, our study indicated that the number of regional lymph nodes examined was an independent risk factor, separate from the other prognostic factors included in the Cox model. There is a correlation between the prognosis of CRC and the number of regional lymph nodes examined. In a study conducted by Murphy et al. [42], it was discovered that there was a notable disparity in the 5-year survival rate between patients who had less than nine regional lymph nodes examined and those who had more than ten (69.4% vs 87.6%, *p* = 0.001). Chang et al. [43] discovered that stage II patients who had less than 11 lymph nodes examined had a 5-year overall survival rate of 73%, those with 11–20 lymph nodes examined had a rate of 80%, and those with more than 20 lymph nodes examined had a rate of 87% (*p* = 0.001). Furthermore, our study indicated that the AUC values of the training cohort and the C-index of the competing risk model exhibited a tendency to increase at 1 year, 3 years, and 5 years. This suggests that the model’s predictive performance and accuracy improved over time, particularly at the 5-year mark. This improvement may be attributed to the mOS of the entire population.

Certain variables included in other scoring systems or prognostic studies were not included in our analyses, such as the number of hepatic metastases. Jang et al. showed that OS in patients with 1–2 CRLM nodules was not statistically different from that in patients with 3–8 CRLM nodules [39]. The advent of modern treatment modalities may explain the similar survival outcomes independent of the number of liver metastases [15]. In recent years, molecular pathological features and gene mutations status (e.g., KRAS, BRAF, NRAS, TP53, microsatellite status, tumor mutation burden and immune checkpoint), and chemotherapy regimen have been shown to correlate with therapeutic response and survival outcomes [44, 45]. Chemotherapy regimens containing oxaliplatin or irinotecan, along with anti-EGFR for tumors with wild-type RAS/BRAF or anti-VEGF for tumors with RAS/BRAF mutations, were successful in enhancing the rate of tumor removal and improving overall survival [46]. The three-agent irinotecan-based chemotherapy regimen (FOLFIRI) has been shown to increase the rate of successful surgery compared to the two-agent regimen. However, it is also associated with a higher incidence of hazardous side effects [47–49].

Our study has the following advantages: (i) First, the existing prognostic models for CRC have encompassed various patient groups, but little focus has been given to the prognosis of CRLM patients who undergo preoperative chemotherapy followed by surgery. This study aims to develop a prognostic model specifically for this group of patients. (ii) Previous research indicates that only about 15% to 25% of CRC patients have combined liver metastases at the time of diagnosis, and the percentage of CRLM patients who can receive preoperative chemotherapy combined with surgical is even lower [50–52]. The enormous sample size from the SEER database allowed us to investigate potential risk factors and construct reasonably accurate predictive models. (iii) The SEER database is highly accurate and objective, which helps to minimize selection bias. In addition, the study included data from 102 patients for external validation, providing further confirmation of the model’s accuracy. (iv) K–M survival analysis method considers competing events as censored events. This can impact the accuracy of survival outcome estimations [53, 54]. In this study, two models were employed: the Cox proportional risk regression model and the competing risk regression model. The objective was to investigate the influence of multifactorial and non-oncological factors on the outcomes in a more comprehensive manner. (v) The study conducted a comparison with the TNM staging system and acquired data from three cohorts of the same system. When compared to the TNM staging approach, the model provided a higher C-index in all three cohorts.

The study we conducted exhibits the subsequent deficiencies: (i) Certain variables, such as treatment regimen, molecular pathological features, and immunotherapeutic markers, were not included in the study due to restrictions imposed by the SEER database and the economic and other objective conditions of the patients in the external validation cohort. (ii) Disparities in race and variations in treatment guidelines contribute to an inherent imbalance in the initial data of patients. Therefore, large-scale, prospective studies are still necessary to generate a higher level of evidence that can aid in clinical decision-making.

## Conclusion

Our findings indicate that age, N stage, perineural invasion, tumor deposit, and regional lymph node metastasis are independent risk factors of Cox proportional risk regression analysis, that can predict the prognosis of CRLM patients who underwent preoperative chemotherapy and surgery. After adjusting for competing risk events, the variables that remained as independent risk factors for cancer-specific death were the number of regional lymph nodes examined, N stage, perineural invasion, tumor deposit, and number of positive regional lymph nodes. Finally, our study discovered that the patients’ mortality risk, as determined by the competing risk model, was lower compared to the Cox proportional risk model, which demonstrated a more precise predictive ability for the risk of cancer-related death. However, further analysis using extensive datasets is necessary to validate this observation.

## Supplementary Information

Below is the link to the electronic supplementary material.

Supplementary file1 (DOCX 17 KB)

## Data Availability

The datasets used and/or analyzed during the current study are available from the corresponding author on reasonable request.
