# Supplementary material for: Constructing a prognostic model for colorectal cancer with synchronous liver metastases after preoperative chemotherapy: a study based on SEER and an external validation cohort
Source: Clin Transl Oncol. 2024 Jun 4;26(12):3169–90. doi: 10.1007/s12094-024-03513-5 (PMC11564222; doi:10.1007/s12094-024-03513-5)
Supplement: Supplementary file 1 — Supplementary file1 (DOCX 17 KB) [file 12094_2024_3513_MOESM1_ESM.docx]

**Supplemental Table 1. Year of diagnosis of patients in the SEER and QDU cohort**

| **Year** | **total SEER** | | **QDU cohort** | |
| --- | --- | --- | --- | --- |
|  | **n** | **%** | **n** | **%** |
| **2010** | **44** | 6.95 | 0 | 0.00 |
| **2011** | **65** | 10.27 | 1 | 0.98 |
| **2012** | **51** | 8.06 | 5 | 4.90 |
| **2013** | **48** | 7.58 | 4 | 3.92 |
| **2014** | **70** | 11.06 | 16 | 15.69 |
| **2015** | **73** | 11.53 | 10 | 9.80 |
| **2016** | **72** | 11.37 | 10 | 9.80 |
| **2017** | **86** | 13.59 | 9 | 8.82 |
| **2018** | **68** | 10.74 | 11 | 10.78 |
| **2019** | **56** | 8.85 | 8 | 7.84 |
| **2020** | **0** | 0.00 | 16 | 15.69 |
| **2021** | **0** | 0.00 | 12 | 11.76 |
